# Anti-tumour activity of deer growing antlers and its potential applications in the treatment of malignant gliomas

**DOI:** 10.1038/s41598-020-79779-w

**Published:** 2021-01-08

**Authors:** Louis Chonco, Tomás Landete-Castillejos, Gemma Serrano-Heras, Martina Pérez Serrano, Francisco Javier Pérez-Barbería, Carlos González-Armesto, Andrés García, Carlos de Cabo, Jose Manuel Lorenzo, Chunyi Li, Tomás Segura

**Affiliations:** 1 Research Unit, Complejo Hospitalario Universitario de Albacete, 02006 Albacete, Spain; 2 Animal Science Techniques Applied to Wildlife Management Research Group, Instituto de Investigación en Recursos Cinegéticos, Universidad de Castilla-La Mancha, 02071 Albacete, Spain; 3 Sección de Recursos Cinegéticos y Ganaderos, Instituto de Desarrollo Regional, Universidad de Castilla-La Mancha, 02071 Albacete, Spain; 4 Departamento de Ciencia y Tecnología Agroforestal y Genética, Escuela Técnica Superior de Ingenieros Agrónomos y Montes, Universidad de Castilla-La Mancha, 02071 Albacete, Spain; 5 Research Department, Neuropsychopharmacology Unit, Complejo Hospitalario Universitario de Albacete, Albacete, Spain; 6 Centro Tecnológico de la Carne de Galicia, rúa Galicia n° 4, Parque Tecnológico de Galicia, San Cibrao das Viñas, 32900 Ourense, Spain; 7 Área de Tecnología de los Alimentos, Facultad de Ciencias de Ourense, Universidad de Vigo, 32004 Ourense, Spain; 8 Institute of Antler Science and Product Technology, Changchun Sci-Tech University, Corner of Pudong Road and Suzhou Bei Street, Changchun, Jilin People’s Republic of China; 9 Department of Neurology, Complejo Hospitalario Universitario de Albacete, 02006 Albacete, Spain

**Keywords:** Cancer therapeutic resistance, Cell death

## Abstract

A recent study showed that antlers have evolved a high rate of growth due to the expression of proto-oncogenes and that they have also evolved to express several tumour suppressor genes to control the risk of cancer. This may explain why deer antler velvet (DAV) extract shows anti-tumour activity. The fast growth of antler innervation through the velvet in close association to blood vessels provides a unique environment to study the fast but non-cancerous proliferation of heterogeneous cell populations. We set out to study the anti-cancer effect of DAV in glioblastoma (GB) cell lines in comparison with temozolomide, a chemotherapeutic drug used to treat high-grade brain tumours. Here we report, for the first time, that DAV extract from the tip, but not from mid-parts of the antler, exhibits an anti-tumour effect in GB cell lines (T98G and A172) while being non-toxic in non-cancerous cell lines (HEK293 and HACAT). In T98G cells, DAV treatment showed reduced proliferation (37.5%) and colony-formation capacity (84%), inhibited migration (39%), induced changes in cell cycle progression, and promoted apoptosis. The anticancer activity of DAV extract as demonstrated by these results may provide a new therapeutic strategy for GB treatment.

## Introduction

Antlers are the only mammalian structure that regenerates each year^1, 2^. They can reach weights of more than 15 kg and lengths of more than 80 cm. This corresponds to a daily growth rate of 1–3 cm in length in the antler tips and tines^3^, and the daily production of more than 20 cm^2^ of skin in the tip, a rate much higher than cancer^2, 4^. Despite this, only a few cases of bone tumours have been reported in antlers^5, 6^ and it has been suggested that developing antlers are particularly resistant to tumour formation^5, 7^. A recent study^8^ has shown that antlers have evolved such fast growth rates due to high elevated proto-oncogenes expression. Such gene expression profiles of antlers had a higher correlation with osteosarcoma (*r* = 0.67–0.78) than to normal growth in bone tissues (*r* = 0.33–0.47). Despite the expression of proto-oncogenes, these and other studies have reported that Cervids also express several tumour suppression genes, especially cofactor and regulator genes of *TP53* gene that promote a strong regulation between the relation of antler growth and inhibition of oncogenesis^8, 9^. The cell growth regulators required for controlled rapid antler regeneration may be active in deer antler velvet (DAV) extract, thereby reducing tumour formation in human or mouse models^10, 11^. This may be one of the reasons behind the use of DAV in Traditional Chinese Medicine (TCM) for over 2000 years where a variety of therapeutical properties have been claimed, including anti-cancer effects, improvement of the immune system, physical strength, and sexual function^12, 13^. Nevertheless, while TCM practitioners use a century-old method involving a wide range of plant, animal, and mineral ingredients, only in the twenty-first century scientific studies are starting to prove some of the claimed properties^14, 15^.

The most prominent bioactive components of velvet antlers from red deer (*Cervus elaphus*), sika deer (*Cervus nippon*), white-tailed deer (*Odocoileus virginianus*), and elk (*Cervus canadensis*) are considered to be their amino acids, polypeptides, and proteins^10^. The DAV extract has been considered as a candidate for inhibiting cancer growth. Accordingly, Fraser et al.^16^ concluded that DAV supplementation decreased the grade and metastasis of azoxymethane-induced colon cancer in male rats. Hu et al*.*^17^ showed a reduction in telomerase gene expression and cell cycle arrest with a new monomeric peptide that effectively inhibited breast cancer cell proliferation. Tang et al.^18^ observed a decrease in the expression of prostate-specific antigen (PSA) and, for the first time, the anti-migration activity of DAV on prostate cancer. Yang et al.^19^ showed that DAV extract specifically reduced tumour growth in cell cultures of prostate cancer as effectively as a chemotherapeutic drug (cisplatin), but it promoted growth in non-cancerous (embryonic) cells. Similarly, Tang et al.^20^ found that DAV extract reduced tumour volume and weight as effectively as cisplatin in xenograft mouse models for prostate cancer, and hence, suggested that it may be a potential treatment to reduce metastasis in humans. In their review of antlers, Landete-Castillejos et al.^2^ suggested that, because antlers have a number of fast-growing types of tissues, DAV extract may be effective against many types of cancer.

A potentially interesting cancer type to test the effects of DAV extract is glioblastoma (GB). This cancer, which is characterized by extensive infiltration into the surrounding brain parenchyma^21^, is the more prevalent and aggressive malignant brain tumour. It represents 2% of all cancer cases, yet it is one of the major reasons for morbidity and mortality associated with cancer^22^. The current standard of care for newly diagnosed GB is surgical resection, to an extent that is feasible, followed by adjuvant radiotherapy plus temozolomide (TMZ)^23^. TMZ, an oral alkylating agent, is the only chemotherapeutic agent that has demonstrated clinical efficiency in the treatment of the gliomas together with surgery and radiotherapy^24^. However, after the described standard treatment regimen, GB patients can expect a median survival of 14.6 months^25^, while less than 5% of patients live longer than 5 years. This poor prognosis is due, in part, to therapeutic resistance which results in tumour recurrence^26^. The limited efficiency of TMZ is due to mechanisms of primary and/or acquired resistance^27^. Therefore, there is an important need to develop novel therapies for single or combined administration to overcome these limitations of the current chemotherapeutic drugs in the treatment of cancer, particularly those with little or no side effects. Alternative therapy could be based on the use of DAV extract. Additionally, the study of the anti-tumour effects may also identify potential targets and processes for the development of pharmaceutical products in the longer term.

To date, no studies have been done to investigate the use of DAV extract in GB development. Therefore, we performed a compilation of preclinical studies, cell proliferation and migration assays, and cell cycle and apoptosis analyses to assess the anticancer activity of DAV extracts in GB cell lines. Because antlers grow from the tip downwards, where the expression of proto-oncogenes and anti-tumour genes should be greater, we expected that the tip of the antler would show greater anti-tumour activity compared to the other antler parts. In this study, we tested the middle part of the antler as a control.

## Results

### Gross characteristics of DAV extract

**Figure 1:**
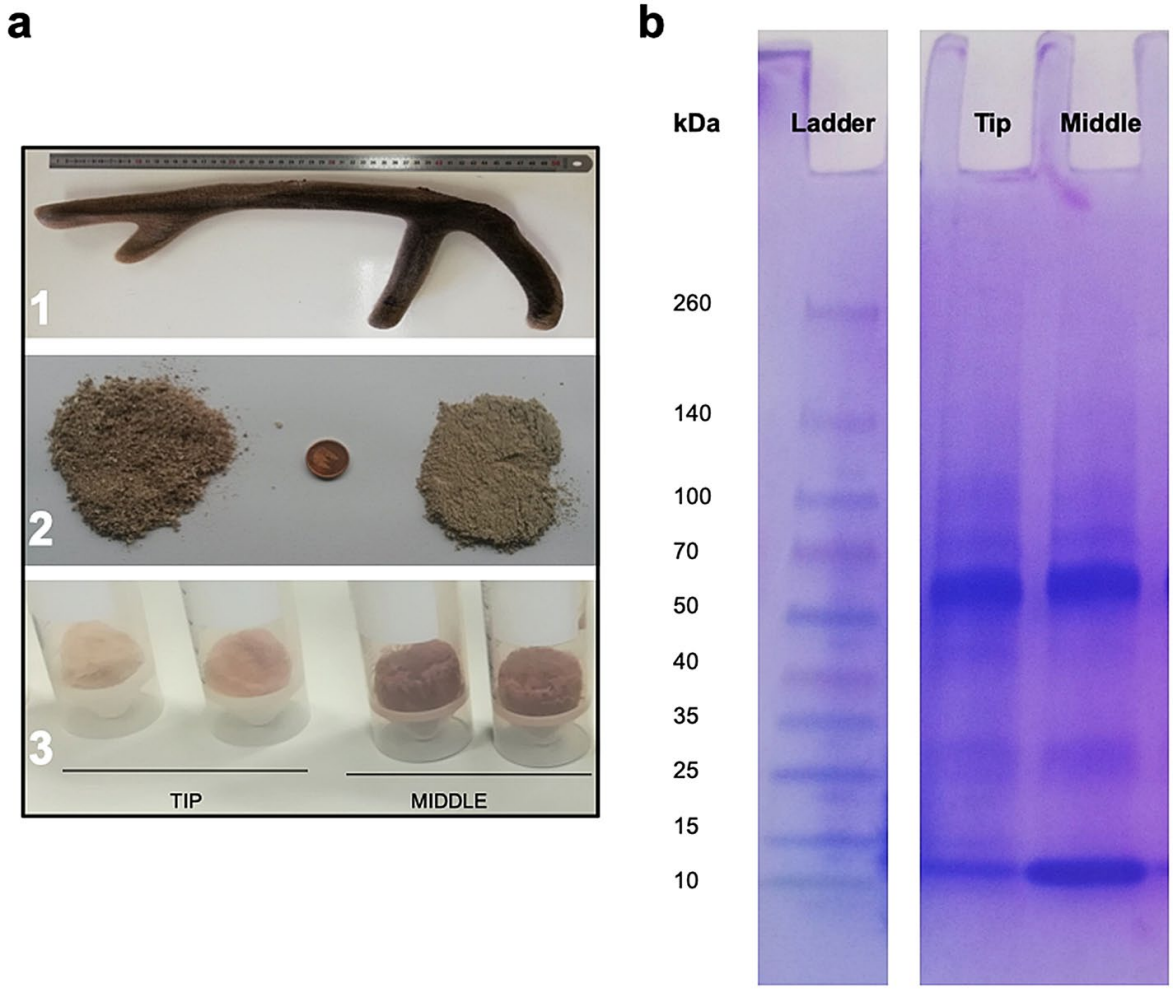
Deer antler velvet (DAV) extract. (**a**) 1. Example of a red deer antler of 2 years old in the optimum phase of growth to be used for the extraction of bioactive products; 2. Tip (2.5 cm) and middle sections (5 cm each) are milled (left) and pulverized (right) to obtain a DAV powder; 3. DAV extract from the tip and middle sections after the freeze-dried process. (**b**) Proteins analyzed by SDS-PAGE on a 4–15% precast polyacrylamide gel (cropped gel. Full-length gel is included in Supplementary Information).

The DAV extract was obtained from the powder of different portions of the antler (tip or middle part), without skinning the portion or choosing particular tissues within it. Figure 1a shows the appearance of dried DAV powder after antler portions were freeze-dried and then ground to a powder (in contrast to TCM processing, which involves steam or brief boiling, often repeatedly). The DAV protein content was 45% in the tip and 65% in the middle using the Bicinchoninic Acid (BCA) method. The composition and size of water-soluble proteins from the tip and middle portions mainly ranged from 100 to 10 kDa by Sodium Dodecyl Sulfate Poly-Acrylamide Gel Electrophoresis (SDS-PAGE) (Fig. 1b).

### Reduction of GB cell proliferation by DAV

**Figure 2:**
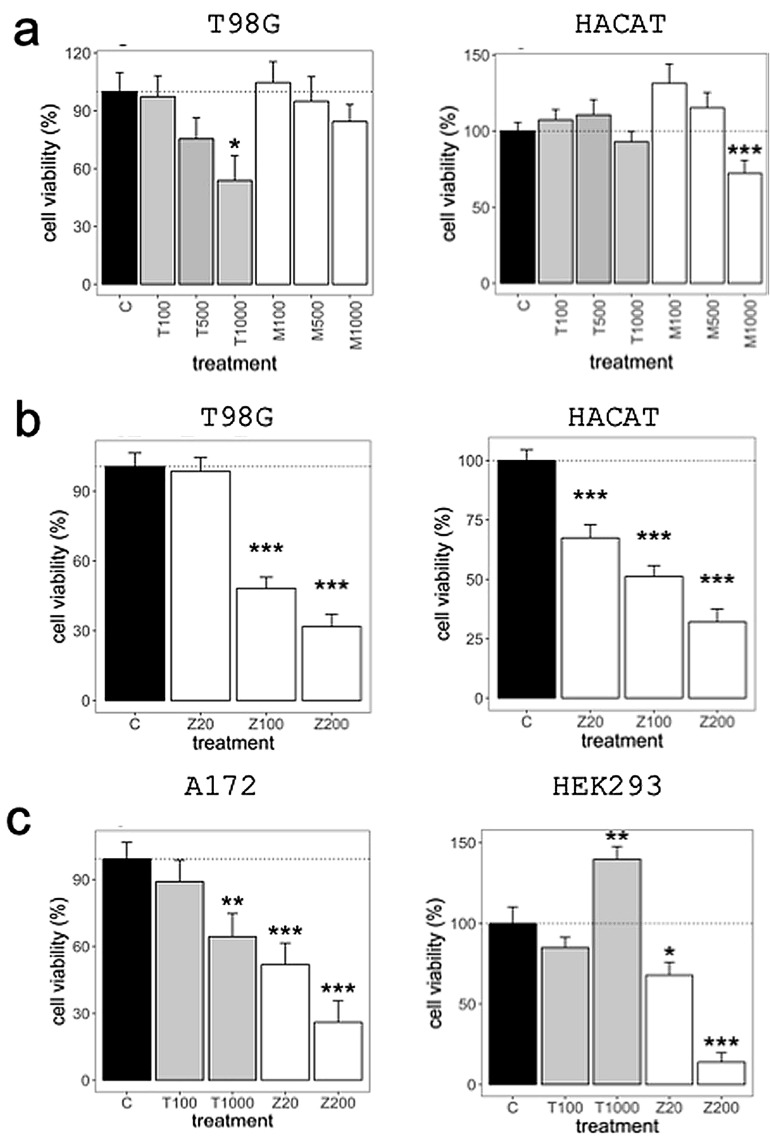
Cell viability assay. (**a**) Cytotoxicity in T98G cell line (left) and HACAT non-cancerous cell line (right) following 72 h treatment with Tip and Middle extracts from the same DAV. (**b**) Dose-dependent representation for T98G (left) and HACAT (right) cell lines following 72 h treatment with TMZ. (**c**) Cytotoxicity in A172 (left) and HEK293 (right) cell lines following 72 h treatment with DAV extract and TMZ. Values are shown in μg/mL. *T *tip portion, *M *middle portion, *Z *TMZ. Linear mixed models to assess the effects of treatments were used. Data are means ± standard error of the mean (SEM) (n = 3). *P*-value of the significant pairwise comparison against control (^+^*P* = 0.1–0.05, *P = 0.05–0.01, **P = 0.01–0.001, ***P ≤ 0.001).

As indicated in our hypothesis, we sought to confirm that DAV extracts can reduce tumour proliferation in GB cell lines. In the MTT assay (a colorimetric assay for assessing cell metabolic activity), the T98G cells treated with DAV extracts from the tip showed a reduced proliferation capacity in a dose-dependent manner, while no toxicity was found in the HACAT cell line (Fig. 2a). Among the 12 different tips used in this study, T98G cells treated with tip portions for 72 h showed an IC50 value (the half-maximal inhibitory concentration) of approximately 1 mg/mL, while middle portions had no reduced cell viability (*P* > 0.05; see Supplementary Table S1 online). Since the results showed greater activity with the DAV extract from the tip, this portion was selected for further experiments, discarding the middle parts for labour- and cost-effectiveness. The treatment of T98G and HACAT cell lines with TMZ showed a dose-dependent toxicity leading to an IC50 of approximately 0.1 mg/mL (500 µM) (*P* < 0.001; Fig. 2b). Moreover, A172 GB cell line treated with DAV extract at 1 mg/mL for 72 h reduced their proliferation capacity, while DAV extract exhibited no toxicity in the HEK293 cell line (*P* < 0.01; Fig. 2c). Notably, DAV extract at 1 mg/mL increased cell proliferation in the HEK293 cell line (*P* < 0.01; Fig. 2c). The A172 cell line, a TMZ-sensitive cell line, showed a 40% inhibition with TMZ at 0.02 mg/mL (*P* < 0.001), while the T98G TMZ-resistant cell line did not (*P* > 0.05). No reduction of cell proliferation was observed in any cell line treated for 24 h with DAV extract from the tip (*P* > 0.05; see Supplementary Fig. S1 online).

**Figure 3:**
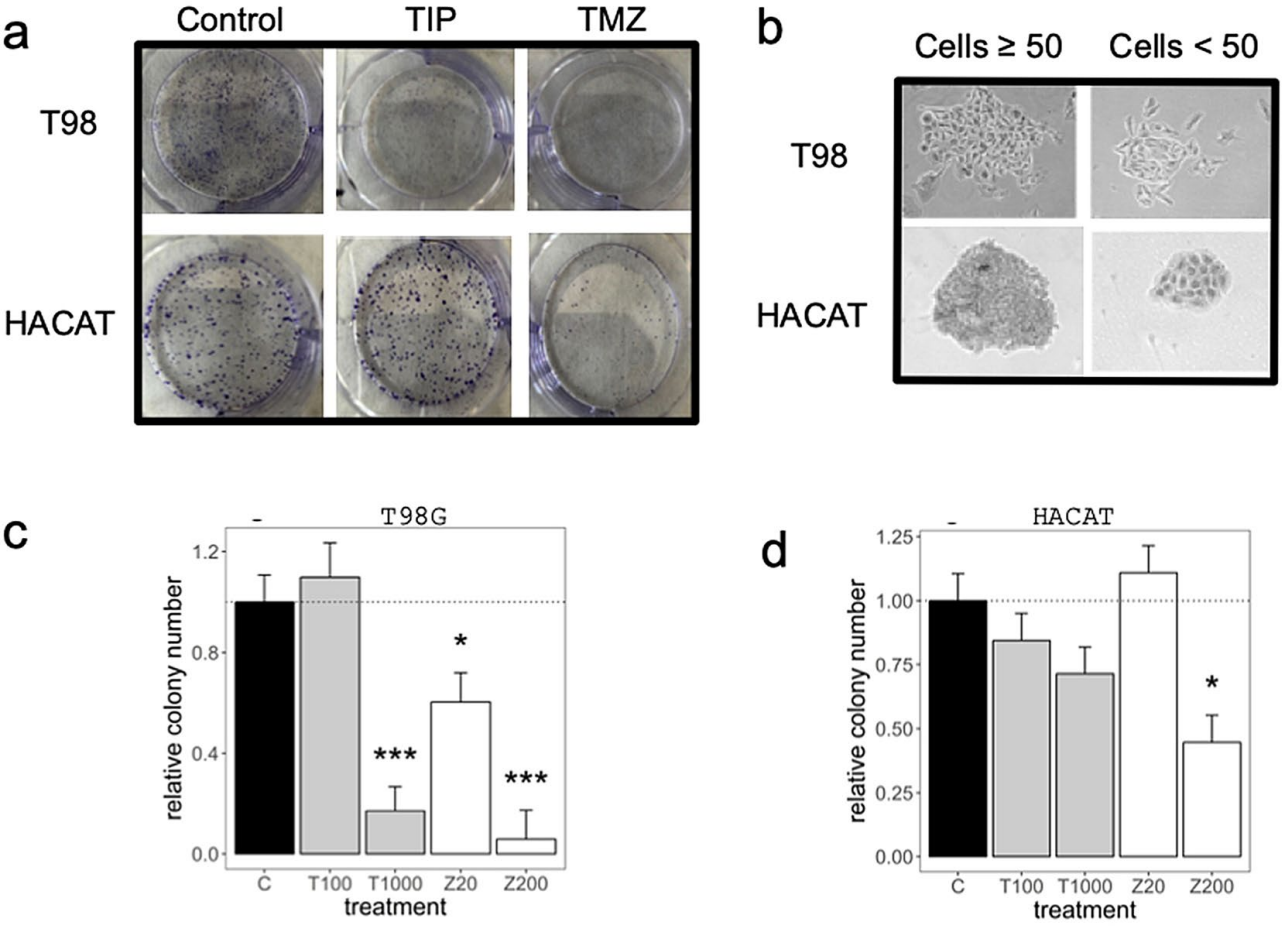
Tumor clonogenic assay (TCA). (**a**) TCA was performed in a 6-well plate, with clones produced by T98G and HACAT cells at day 12 following treatment with DAV and TMZ at 1 mg/mL and 0.2 mg/mL, respectively. (**b**) The number of colonies containing ≥ 50 cells was counted under a microscope. (**c**,**d**) Relative colony number was represented for T98G and HACAT cells, respectively. Values are shown in μg/mL. Linear mixed models to assess the effects of treatments were used as described in Fig. 1. Data are means ± SEM (n = 2).

Figure 3 shows the results of the Tumour Clonogenic Assay (TCA) which evaluates the ability of a single cell to grow into a colony. The colony-formation capacity in T98G cells was reduced by 84% with DAV extract at 1 mg/mL (*P* < 0.01) indicating that it could inhibit T98G cell growth. However, HACAT cells did not show a reduction at the same concentration (*P* > 0.05).

These viability assays showed that DAV extract from the tip at 1 mg/mL inhibited T98G and A172 GB cell proliferation with a non-toxic effect in both the HEK293 and HACAT non-cancerous cells.

### Scratch Assay

**Figure 4:**
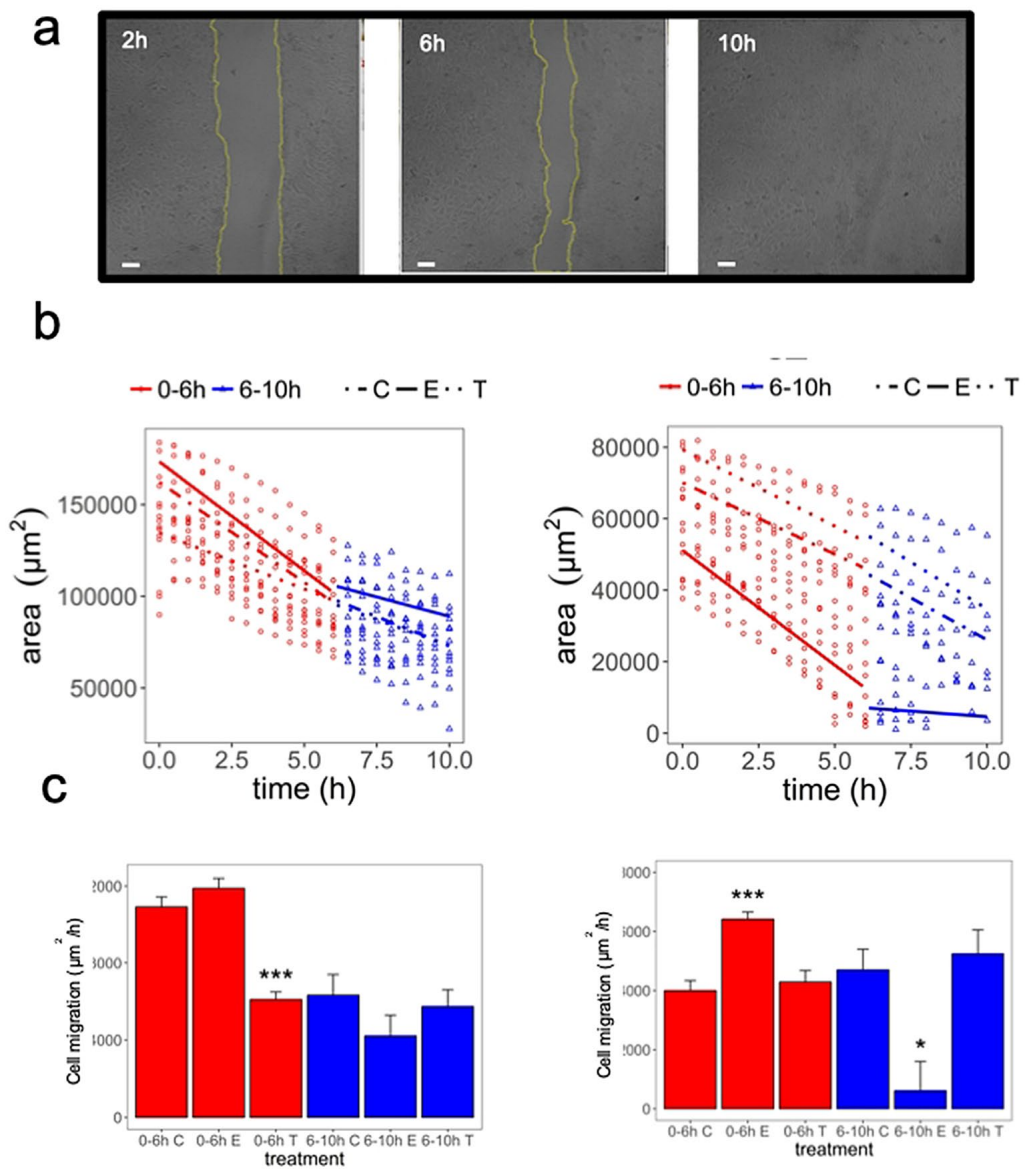
Scratch assay. (**a**) HACAT cell images from a wound-healing experiment at different time points. As an example, EGF treated cells at 10 ng/mL are shown at 2, 6, and 10 h. Scale bar = 100 μm. (**b**) T98G and HACAT cell images in a time series were analyzed for gap area over time. Linear mixed regression to model the rates of wound healing across time is shown. (**c**) The cell migration rate for T98G and HACAT cells is shown. Values in brackets stand for compound concentration in mg/mL. *C *control, *T *tip portion, *E *EGF. Data are means ± SEM (n = 2). *P*-value of the significant pairwise comparison against control (^+^*P* = 0.1–0.05, *P = 0.05–0.01, **P = 0.01–0.001, ***P ≤ 0.001).

In the scratch assay (which assesses the coordinated movement of a cell population), HACAT cell monolayers which were treated with Epidermal Growth Factor (EGF) at 10 ng/mL, exhibited a complete wound healing at 10 h (Fig. 4a). Figure 4b,c showed the area reduction as a function of time for T98G and HACAT cells treated with EGF (10 ng/mL) and DAV extract (1 mg/mL). Rates of wound healing over different time intervals were significantly different between treatments. We identified two cell migration rates from 0–6 h and 6–10 h in T98G and HACAT cells (Fig. 4b,c and Supplementary Table S2 online). Figure 4c shows the cell sheet migration rate (μm^2^/h) at which the cells collectively move into the gap. The T98G cells treated with DAV extract at 1 mg/mL showed a decrease of 39% as compared to the control group from 0–6 h (*P* < 0.001), while HACAT cells treated with DAV at 1 mg/mL did not. Nevertheless, the HACAT cells treated with EGF at 10 ng/mL showed an increase in cell migration (*P* < 0.001) which was not observed in T98G cells. Overall, the bioactive compounds in 1 mg/mL DAV extract showed an inhibition of the cell migration rate in the T98G but not in HACAT cells (*P* > 0.05).

### Effect of DAV on cell cycle profile

**Figure 5:**
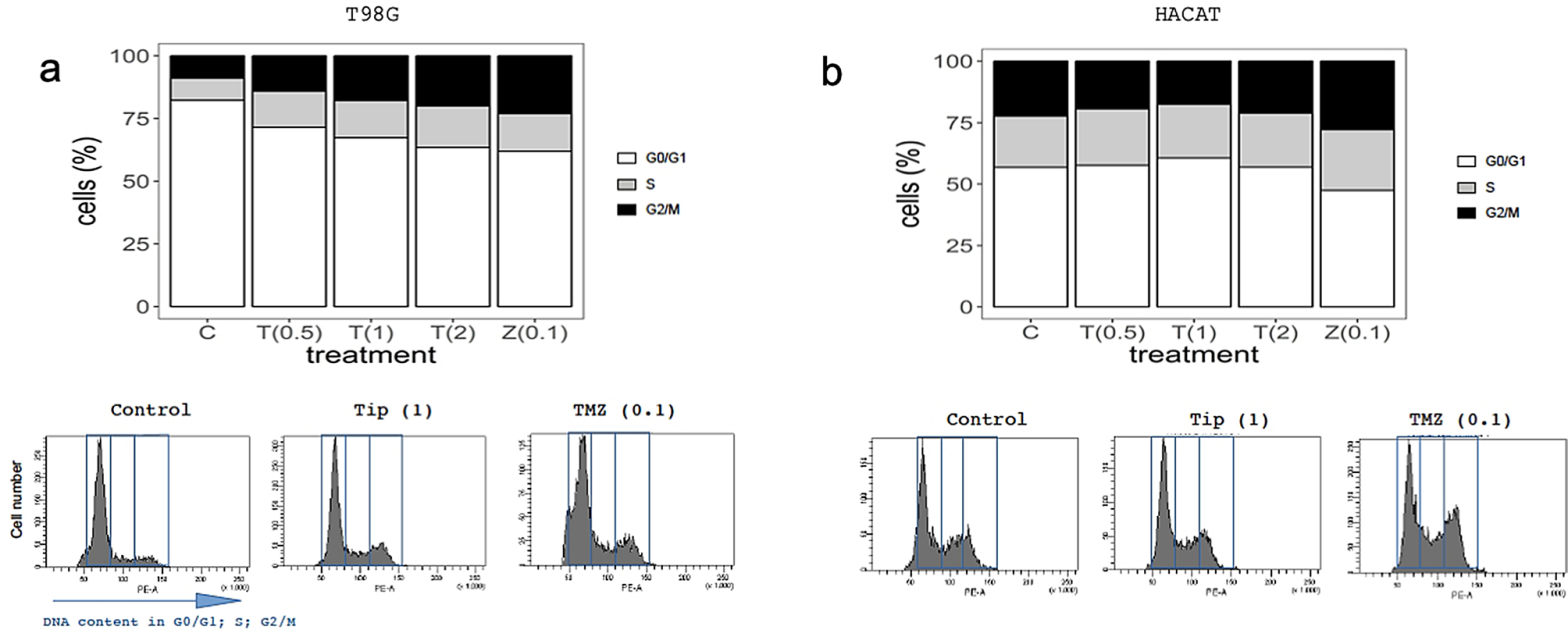
Cell cycle study. (**a**) Bar graphs illustrate results of T98G cell cycle analysis, indicating the percentage of cells in the G0/G1, S, and G2/M cell cycle phases following 24 h treatment with concentrations of 0.5, 1, and 2 mg/mL DAV and with TMZ at 0.1 mg/mL. Below, representative histogram plots of cell cycle distribution showing DNA content in each phase. (**b**) The same analysis as (**a**) is shown for HACAT cells. Values in brackets stand for compound concentration in mg/mL. Linear mixed models to assess the effects of treatments were used as described in Fig. 1. Data are means ± SEM (n = 2).

The DAV extract induced changes in GB cell cycle progression (Fig. 5a). Flow cytometry was performed on T98G cells to assess the cell cycle profile, as described in the Methods section and Supplementary Fig. S2 online. As shown in Fig. 5a, bar graphs represent the results of T98G cell cycle analysis and representative histogram plots were highlighted below. The percentage of T98G cells in each cell cycle phase was quantified and compared to the control group (see Supplementary Fig. S2 online). Differences in T98G cells were obtained following 24 h treatment with concentrations of 0.5, 1, and 2 mg/mL DAV. The percentages differences of DAV extract at 1 mg/mL were 18.5% G0/G1 phase reduction (*P* < 0.05), 69% S phase increase (*P* < 0.05), and 54% G2/M phase increase (*P* > 0.05). No differences were obtained on HACAT cells following 24 h treatment with concentrations of 0.5, 1, and 2 mg/mL DAV (*P* > 0.05; Fig. 5b). Therefore, DAV extract altered the percentage of T98G cells in every phase and caused high populations of T98G cells to be arrested in the S and G2/M phases.

### Induction of apoptosis following DAV treatment

**Figure 6:**
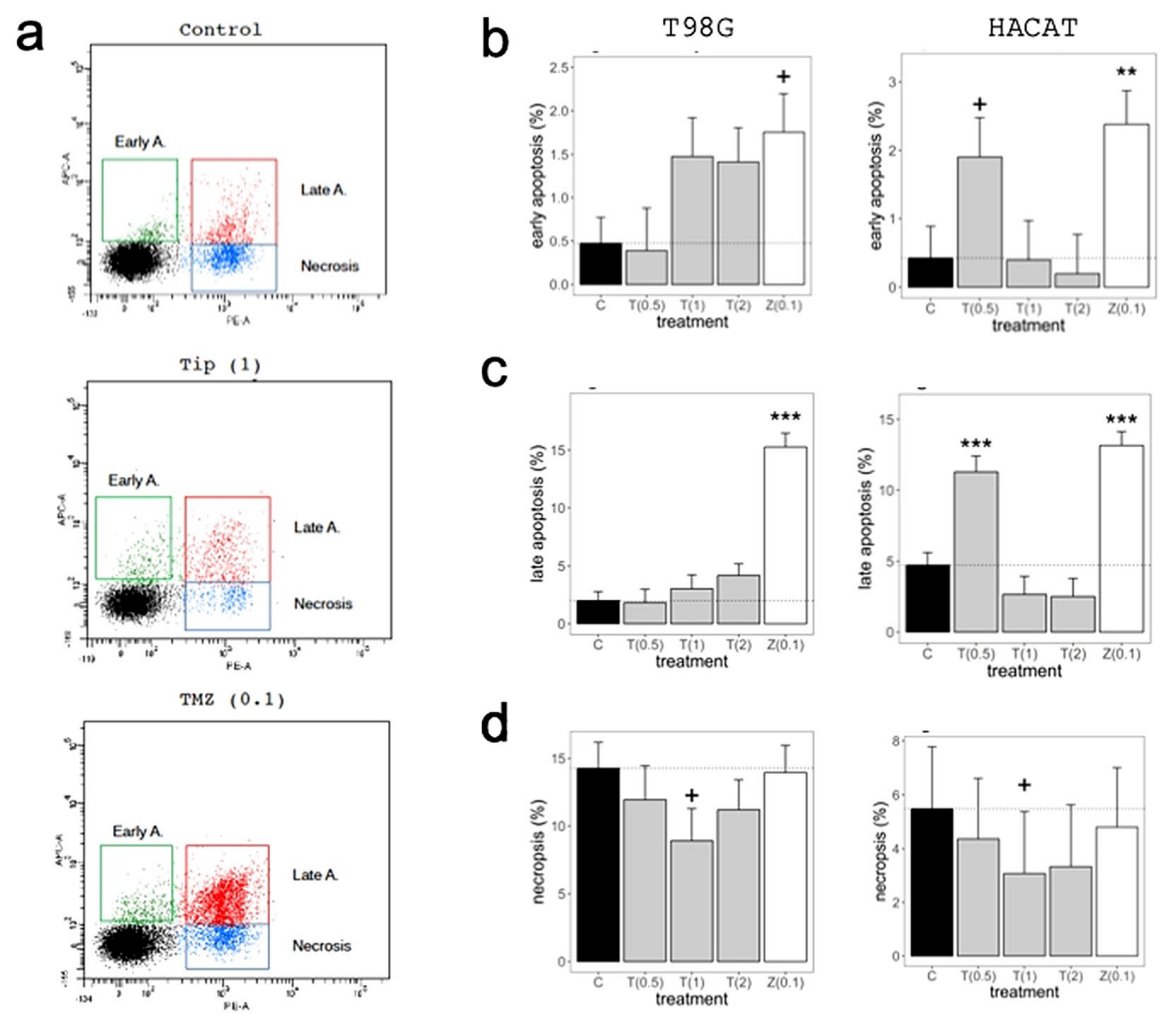
The effects of DAV in T98G and HACAT cell apoptosis were analyzed by flow cytometry at 72 h. (**a**) Early apoptotic (Annexin V-positive, PI-negative), late apoptotic (Annexin V-positive and PI-positive), and necrotic cells (Annexin V-negative and PI-positive) were included in cell death determinations by flow cytometry at 72 h. An example of T98G cells is shown. (**b**) Percentage of T98 early apoptotic cells (left) and HACAT early apoptotic cells (right) following treatment with concentrations of 0.5, 1, and 2 mg/mL DAV and with TMZ at 0.1 mg/mL. (**c**,**d**) The same analysis as (**a**) is shown for late apoptotic cells and necrotic cells, respectively. Values in brackets stand for compound concentration in mg/mL. Linear mixed models to assess the effects of treatments were used as described in Fig. 1. Data are means ± SEM (n = 2).

The effects of DAV extract in T98G and HACAT cell apoptosis were analyzed by flow cytometry at 72 h (Fig. 6a). The number of T98G early apoptotic cells were three times higher than the control group following treatment with 1 mg/mL and 2 mg/mL DAV extract, although results did not achieve statistical significance (both with *P* > 0.05; Fig. 6b). Moreover, results showed a trend (which also failed to achieve statistical significance) of a greater number of late apoptotic cells in a dose-dependent manner compared to the control group following treatment with DAV extract (*P* > 0.05; Fig. 6c). At these higher concentrations, DAV extract did not affect HACAT cell percentages as compared to the control group (*P* > 0.05), but at the lower 0.5 mg/mL, DAV induced an increase of early and late apoptotic cell percentages in HACAT cells (*P* < 0.1 and *P* < 0.001, respectively). Furthermore, the number of necrotic cells also decreased at 1 mg/mL in T98G and HACAT cells (both with *P* < 0.1; Fig. 6d). TMZ at 0.1 mg/mL showed a significant increase in early and late apoptotic cells in T98G and HACAT cell lines. No differences were observed following 24 h treatment at 1 mg/mL DAV extract in T98G cells (see Supplementary Fig. S3 online). These results showed that DAV extract could induce both early and late apoptosis in T98G cells at 72 h. The DAV anti-tumour effects shown in T98G cells may be at least partly explained by a small induction of cell apoptosis.

## Discussion

This study provides the first evidence that bioactive compounds from DAV extract have anti-cancer effects in GB cell cultures, with a lack of cytotoxicity in non-cancerous cells. These results support the hypothesis by Wang et al.^8^ that DAV evolved to express several tumour suppressor proteins, and also that of Landete-Castillejos et al.^2^ that the bioactive molecules derived from tumour suppressor genes should show a broad spectrum activity against different types of cancer, GB in our case. The bioactive factors derived from tumour suppressor genes and cell growth regulators, required for controlled rapid antler regeneration, may remain active in DAV extract. Further studies that investigate alternative extraction methods may show even greater anti-cancer activities of DAV. Such possibilities, coupled with the results of this study, therefore open a whole new promising field in cancer research.

Wang et al.^8^ hypothesized (and provided evidence) that, to control the risk of cancer, deer also express tumour suppressor genes (such as *TP53*)*.* Thus, in addition to proto-oncogenes specific to osteosarcoma, deer antler may also contain factors that specifically suppress cancers of the other fast-growing tissues in the antler such as skin, nerves, or blood vessels. Landete-Castillejos et al.^2^ postulated that the bioactive molecules of DAV extract may be active against a broad spectrum of cancers, as we have found in this study for the case of GB. The basis of the hypothesis of Wang et al.^8^ explains why previous researchers had found that DAV extract showed anti-cancer properties in cancers very different from the tissues involved in the growing antler, such as the human prostate^18–20^. These researchers, and all those published to date, used commercially available DAV extract from the whole antler^17, 28, 29^. However, the fact that antlers grow from the tip of the beam and tines^30–32^ led us to postulate that the expression of tumour suppressor genes and related bioactive molecules, should be greater in the tip compared to other parts of the antler. Tang et al.^18^ tested different parts of growing antlers and found that the DAV tip showed the highest anti-prostate cancer cytotoxicity.

T98G cells treated with tips for 72 h showed an IC50 value of approximately 1 mg/mL, while the middle portions did not reduce cell viability significantly. This supports the expected results that tips are more effective against cancer compared to other parts of the antler. The TMZ showed greater toxicity against GB, as it has an IC50 of 0.1 mg/mL (500 µM), which was also demonstrated by Towner et al.^33^. However, TMZ showed a non-specific inhibition as it inhibited cancerous and non-cancerous cells similarly, with inhibition rates above 70% in HEK293 and HACAT cell lines (0.2 mg/mL). A172 TMZ-sensitive cell line showed an inhibition with 0.02 mg/mL while the T98G TMZ-resistant cell line did not. Not only DAV extract was not toxic against non-cancerous cells, but it increased HEK293 cell proliferation at 72 h. If DAV extract were to be used as a therapy for the treatment of GB in combination with TMZ, it is likely that the growth factors contained in the extract might benefit surrounding normal tissues, deteriorated by the non-specific toxicity of TMZ treatment. These results highlight the cell-population specificity of the bioactive compounds and the balance between tumour suppressor proteins and growth factors, as described previously by Yang et al.^19^.

The TCA can predict sensitivity or resistance toward clinically used agents. For labour efficiency, we focused on T98G and HACAT cell lines. The colony-formation capacity in T98G cells was significantly reduced by DAV extract at 1 mg/mL while HACAT cells did not show a reduction at the same concentration. Therefore, DAV has therapeutic potential for GB treatment, because targeting a clonogenic/tumour-initiating/stem cell-like subset of cancer cells is thought to be essential for successful cancer therapy^34^.

Sui et al.^10^ reported the positive effects of DAV extract on skin wound healing in rats due to its growth factors including insulin-like growth factor-1 (IGF-1), nerve growth factor (NGF), and EGF. The DAV extract at 1 mg/mL showed an inhibition of the cell migration rate in T98G but not in HACAT cells.

An additional assay was carried out to assess the changes in cell cycle progression of GB cancer cells by DAV extract. Our results showed that DAV induced changes in GB cell cycle progression. In particular, our results showed that DAV extract and TMZ altered the percentage of T98G cells in each phase, decreasing the number of cells in the G0/G1 phase causing T98G cells to be arrested in the S and G2/M phases. For DAV extract, the effect is dose-dependent, so that it is nearly more similar to TMZ at 1 and 2 mg/mL as compared with 0.5 mg/mL. Wang et al.^35^ showed that TMZ-induced the G2/M arrest in T98G cells and HACAT keratinocytes on day 1 only, as we found also for TMZ. However, DAV extract did not affect HACAT cells. Hu et al.^17^ found that a monomeric peptide from antler plate polypeptide of sika deer also altered cancer cell cycle progression (in a breast cancer cell line), but in their case DAV peptide stopped cells in the G0/G1 phase and inhibited DNA synthesis. However, unlike our study, they did not assess effects on non-cancerous cell line controls.

Our results suggest that a small induction of cell apoptosis may at least partially explain the anti-tumor effects of DAV extract against T98G cells. This is because the number of early apoptotic cells was three times higher compared to the control group, although the high variability in our small sample prevented to reach statistical significance. However, no trend was found for HACAT cells. A role for autophagy in chemotherapy-induced cancer cell death must be taken into account and further assays would confirm the underlying mechanism involved^36^.

The only significant increase in both early and late apoptotic HACAT cells was at 0.5 mg/mL DAV extract. We consider these results a surprising effect of low-end concentrations of DAV, because it is paradoxical as it disappears at higher concentrations, and it would be the object of further research. In general, the non-statistical DAV extract trend mentioned above in T98 agrees with the significant anti-cancer effects on prostate cancer cells apoptosis^19^.

Although we did not include DAV extract effects in vivo, which will be assessed in a further study based on published animal studies and clinical trials, the deer antler base causes no severe side effects^37^. A series of in vivo studies suggest that water-soluble extract of deer antler base suppressed the tumour growth in mice with breast cancer and prolonged the survival time of mice inoculated abdominally with sarcoma cells^38^. DAV may show similar anti-GB effects in vivo while being non-toxic for the animal. However, the efficiency of the DAV extract in vivo is reliant upon its bioactive molecules successfully crossing the blood–brain barrier in order to reach the GB.

## Conclusion

In conclusion, our study demonstrates that DAV exerted a cytotoxic effect on GB cells. Cell death might be linked to halting the cell cycle in a brain cancer cell line, and non-significant trends in our small sample size also suggest the activation of at least early apoptosis. DAV showed no adverse-effects in comparison to TMZ chemotherapy treatment. Overall, our data suggest that DAV extract contains bioactive compounds with tumour suppressor properties and might be developed as a valuable therapeutic drug for the treatment of GB.

## Methods

### Antler samples

Antlers were sampled from 12 adult males of red deer which were hunted for other purposes (summer selective shooting to reduce population density) in a deer private game state in Ciudad Real province (38°53′N, 4°17′E). Males were chosen among those who had antlers in a growth stage similar to farm animals at 60 days of age (from our experience in the experimental deer of the UCLM and guidelines from Deer Industry New Zealand^39^). Immediately after death, antlers were cut off with a mechanical saw, and then, kept refrigerated until they were stored frozen at − 20 °C until they could be freeze-dried. They were then divided into portions (tip, about 2.5 cm in the top and middle portions). Each portion was milled until particles were less than 0.18 mm.

### Production of DAV extract and protein quantification

The DAV powder (1 g) was weighed and soaked with 10 mL distilled water. The liquid mixture was incubated at 4 °C overnight with continuous stirring and then centrifuged at 2700*g* for 20 min. The supernatant was freeze-dried and dissolved into 2 mL Phosphate Buffered Saline (PBS, Lonza BioWhittaker). Samples were frozen at − 80 °C and to carry out any further assay, DAV samples were thawed, past through a 0.22 μm filter, and centrifuged at 5600*g* for 3 min. Protein concentration in DAV extracts was determined by the BCA Protein Assay Kit (Sigma-Aldrich). BCA was used to measure the protein concentrations before performing SDS-PAGE and further assays.

### SDS-PAGE

SDS-PAGE is a technique for separating mixtures of proteins based on their size. Briefly, a 4–15% precast polyacrylamide gel (Mini-Protean TGXTM, BIO-RAD) was used in a vertical electrophoresis gel. Tris glycine 1 × and SDS 0.1% was used as the running buffer. 20 μg/sample was loaded in the gel in 25 μL of buffer 1 × (Laemmil Loading buffer, 1 ×). 10 μL of Spectra Multicolor Broad Range Protein Ladder was added in the first well. Gel electrophoresis was conducted for 45 min at 120 V. Coomassie blue staining was performed following the protocol described in Brunelle and Green^40^. The stain solution was filtered through Whatman filter paper grade 1. Both solutions were stored at room temperature.

### Culture media, reagents and cell lines

Two stable malignant GB cell lines were used: (i) T98G, a human GB multiforme cell line resistant to TMZ that expresses high levels of MGMT (O(6)-Methylguanine-DNA methyltransferase) enzyme and MPG (N-Methylpurine-DNA Glycosylase) enzyme. T98G was obtained from the American Type Culture Collection (ATCC CRL-1690, Rockville, MD, USA); and (ii) A172, a human GB cell line sensitive to TMZ that expresses low levels of MGMT and MPG enzymes. A172 was obtained from the American Type Culture Collection (ATCC CRL-1620, Rockville, MD, USA). Furthermore, two stable non-cancerous cell lines were used: (iii) HEK293, a human embryonic kidney cell line (ATCC CRL-1573), and (iv) HACAT, a human keratinocyte cell line (CLS Item number: 300493). T98G and HEK293 cell lines were cultured in Eagle’s Minimum Essential Medium (EMEM) ATCC 30-2003. A172 and HACAT cell lines were cultured in ATCC-formulated Dulbecco's Modified Eagle's Medium (DMEM) ATCC 30-2002. All media were supplemented with 10% Fetal Bovine Serum (FBS), 100 U/mL penicillin, 100 μg/mL streptomycin, and 2 mM l-glutamine and cells were maintained in the cell culture incubator at 37 °C and 5% CO_2_ atmosphere.

### Cell viability assessment

The MTT (3-(4,5-Dimethyl-2-thiazolyl)-2,5-diphenyl-2H-tetrazolium bromide) assay was used to measure cellular metabolic activity as an indicator of cell viability, proliferation, and cytotoxicity. This colorimetric assay is based on the reduction of a yellow tetrazolium salt to purple formazan crystals by metabolically active cells. The viable cells contain NAD(P)H-dependent oxidoreductase enzymes which reduce the MTT to formazan. The insoluble formazan crystals are dissolved using dimethylsulfoxide (DMSO) and the resulting colored solution is quantified by measuring absorbance at 555 nm (690 nm reference filter) using a multiwell plate reader (SPECTROstar Omega spectrophotometer, BMG Labtech). The amount of color produced is directly proportional to the number of viable cells. The cell viability was calculated using the following equation:

$$Cell\;viability\;(\% ) = \frac{Mean\;absorbance\;of\;the\;sample}{{Mean\;absorbance\;of\;the\;control}} \times 100$$

T98G and HEK293 cell lines were seeded in EMEM at 2500 cells/well in a 96-well culture plate (200 μL/well). A172 and HACAT cell lines were seeded in DMEM at 2500 cells/well in a 96-well culture plate (200 μL/well). On the following day, cells were at 30–40% confluence and cell culture medium with 10% FBS was replaced with 2.5% FBS culture medium (control cells) or DAV dilutions (0.1, 0.5, and 1 mg/mL) in 2.5% FBS culture medium. The culture plates were kept for a further 24 h or 72 h in the cell culture incubator. Then, the culture medium was replaced with culture medium (0.5 mg/mL) containing 200 μL of MTT (Sigma) without phenol red. The culture plates were kept for further 40 min in the incubator at 37 °C, 95% humidity and 5% CO_2_. The culture medium was then replaced with 200 µL of DMSO for the solubilization of the formazan crystals.

All experiments were carried out in quadruplicates. Results were plotted as the mean values ± standard error of the mean (SEM) from experiments repeated independently at least twice. The IC50 was estimated by plotting various concentrations of DAV and TMZ versus the percentage of cell viability.

### Tumour clonogenic assay

The TCA or colony-formation capacity assay is an in vitro cell survival assay where the ability of a single cell to grow into a colony following DAV treatment was studied. Briefly, T98G and HACAT cells were plated in 6-well culture plates at a density of 1000 cells/well in EMEM and DMEM cell culture medium with 10% FBS, respectively. On the following day, the medium was replaced with freshly prepared DAV extracts (Tip#2 and Tip#5 at 0.1 and 1 mg/mL) and TMZ at 0.02 and 0.2 mg/mL. After 12 days of incubation at 37 °C, the medium was removed and the cells were washed twice with PBS, fixed 30 min in 0.5% glutaraldehyde solution 25% (Merck), and stained for 20 min in 0.1% crystal violet solution (Merck). The number of colonies containing ≥ 50 cells was counted using a microscope.

### Scratch assay

The scratch assay or wound healing assay has been used in a range of disciplines to study the migration of a cell population. Data was obtained from HACAT and T98G cells grown on plastic 24-well plates (150,000 cells/well) to one-day past confluence and then scratched with a p200 tip (1 mm wound was made in a confluent monolayer). Videos are available as Supplementary Information to observe the gap area for each frame and the cell migration at which the cells collectively move into the gap. The wells were washed once with PBS and then they were treated with 1 mL DAV at 1 mg/mL or 1 mL EGF at 10 ng/mL. All treatments and the controls were prepared in DMEM 0.5% FBS (HACAT) or EMEM 0.5% (T98G). Basal Images (0 h) were taken immediately. Experiments were recorded using a Zeiss LSM710 laser-scanning confocal microscope (Zeiss, Oberkochen, Germany) equipped with a motorized stage and an incubator. Cells were kept at 37 °C in a humid and 5% CO_2_ air-mixture controlled environment. Images were captured every 30 min for 15 h using a 10 × Plan Neofluoar 0.3 NA objective, using a 633 nm laser and a transmitted light PMT detector. Capturing conditions resulted in 512 × 512-pixel images with a 2.07-µm pixel size and 1.58-µs laser dwell time. Time-series images were analyzed for gap area over time, as described in the statistical analysis section.

### Cell cycle and apoptosis/necrosis analysis by flow cytometry

Cell cycle analysis in T98G and HACAT cells was performed in a 6-well plate and cells were seeded at 200,000 cells per well. On the following day, cells at indicated concentrations were treated. After exposure to DAV or TMZ for 24 h in the incubator, cultured cells were collected by pooling together the non-attached and attached cells and incubated in trypsin-EDTA. Cells were then washed twice with cold PBS, fixed in ice-cold 70% ethanol for 30 min and were subsequently washed with PBS + 2% BSA at 2700*g* for 5 min (4 °C). Cell pellets were treated with Propidium iodide/RNAse staining solution (Immunostep S.L., Salamanca, Spain) in the dark for 1 h at 4 °C.

Apoptosis and necrosis detection in T98G and HACAT cells were performed in a 6-well plate. Cells were seeded at a density of 150,000 cells and 100,000 cells to carry out the detection at 24 h or 72 h, respectively. The day after, cells were treated at indicated concentrations. After exposure to DAV or TMZ for 24 h or 72 h in the incubator, cultured cells were collected by pooling together the non-attached and attached cells and incubated in trypsin-EDTA. Cells were then stained with 5 μL of Annexin V-DT-634 (Immunostep S.L., Salamanca, Spain) and 3 μL of Propidium iodide (10 mg/mL) in 300 μL 1 × Binding Buffer (10 mM HEPES, pH 7.4, 140 mM NaOH, 2.5 mM CaCl_2_) for 1 h at room temperature in the dark. Both early apoptotic cells (Annexin V-positive, PI-negative), late apoptotic cells (Annexin V-positive and PI-positive) apoptotic, and necrotic cells (Annexin V-negative and PI-positive) were included in cell death determination.

All cell samples were analyzed using a FACSCanto II flow cytometer (BD Biosciences) and the data analysis was performed using the FACSDiva software v. 6.1.3.

### Statistical analysis

Each of the aims of the study was supported by several assays (experimental block) that varied between 1 and 3, depending on the analytical logistics necessary to accomplish the required treatments, levels, and replicates. We used linear mixed models to assess the effects of treatments (fixed effects) on the different response variables, accounting for the variability between assays within the experimental block as a random effect in the model. Similarly, we used linear mixed regression to model the rates of wound healing across different time intervals (0–10 h) of different cell lines and under different treatments. Video recording across the experiment suggested that there was a change of wound healing rate at time = 6 h, and consequently, we assessed rates of wound healing within two phases, between 0 and 6 h (0–6 h) and between 6 and 10 h (6–10 h). Pertinent comparisons between phases and treatments were carried out for each of the cell lines. An arbitrary number of reference points (between 3 and 8) was used. The advance of cell proliferation was measured on the video images using NIH ImageJ software^41^. Therefore we nested reference points within the treatment and used as random effects in the model.

Some objectives could be carried out in one experimental assay, and in these cases, simple regression analysis was used. For each of the models, we produced a table of the estimates of the coefficients, their standard errors, and *P-*values. Based on these tables a posteriori multiple comparisons of the estimated marginal means of controls against treatment levels were carried out using Dunnett’s test, which was displayed on bar plots together with their standard errors and the *P*-value of the significant pairwise comparison against control (^+^*P* = 0.1–0.05; *P = 0.05–0.01; **P = 0.01–0.001; ***P ≤ 0.001). The contrast was carried out using the R package emmeans^42^. Linear mixed models were implemented using the R software^43^ package lme4^44^ and lmerTest^45^. Since estimating the correct degree of freedom is not possible in linear mixed models, we used the Satterthwaite's degrees of freedom approximation in lmerTest R package^46^. The coefficients of the linear mixed models were calculated using REML, as the estimates are more accurate than using maximum likelihood^47^. The variance explained by the linear mixed model was represented as *R*^2^ marginal (variance accounted for by the fixed effects (*R*^2^_LMM(m)_) and *R*^2^ conditional (variance accounted for by random and fixed effects; *R*^2^_LMM(c)_), following a method developed for linear mixed-effects models^46^ and used in Pérez-Barbería et al.^48^. Graphics were constructed in R software using the ggplot2 package based on The Grammar of Graphics^49^.

### Ethics statement

All methods were carried out in accordance with relevant guidelines and regulations and all in vitro experimental protocols were approved by the research department at Complejo Hospitalario Universitario de Albacete. The antler samples used in this study were obtained from deer hunted for purposes other than experimental ones. Thus, no experimental code of ethics from our university was needed. The slaughter of the hunted animals was regulated by the Regional Hunting Law of Castilla la Mancha^50^, modified by^51^.

## Supplementary Information

Supplementary Video 1.

Supplementary Video 2.

Supplementary Video 3.

Supplementary Video 4.

Supplementary Video 5.

Supplementary Video 6.

Supplementary Information.

## Data Availability

All data generated or analyzed during this study are included in this published article.
