# Supplementary material for: Anti-tumour activity of deer growing antlers and its potential applications in the treatment of malignant gliomas
Source: Sci Rep. 2021 Jan 8;11:42. doi: 10.1038/s41598-020-79779-w (PMC7794318; doi:10.1038/s41598-020-79779-w)
Supplement: Supplementary file 7 — Supplementary Information. [file 41598_2020_79779_MOESM7_ESM.pdf]

Anti-tumour activity of deer growing antlers and its potential applications in the treatment of malignant gliomas

Louis Chonco[1*], Tomás Landete-Castillejos[2,3,4], Gemma Serrano-Heras[1], Martina Pérez Serrano[2,3,4], Javier Pérez-Barbería[2,3,4], Carlos González-Armesto[1], Andrés García[2,3,4], Carlos de Cabo[5], Jose Manuel Lorenzo[6,7], Chunyi Li[8], and Tomás Segura[1,9*]

*CORRESPONDING AUTHORS: Louis Chonco. louischonco@gmail.com; Tomás Segura. tseguram@gmail.com

[1]Research Unit, Complejo Hospitalario Universitario de Albacete, 02006 Albacete, Spain. [2]Animal Science Techniques Applied to Wildlife Management Research Group, Instituto de Investigación en Recursos Cinegéticos, Universidad de Castilla-La Mancha, 02071 Albacete, Spain. [3]Sección de Recursos Cinegéticos y Ganaderos, Instituto de Desarrollo Regional, Universidad de Castilla-La Mancha, 02071 Albacete, Spain. [4]Departamento de Ciencia y Tecnología Agroforestal y Genética, Universidad de Castilla-La Mancha, 02071 Albacete, Spain. [5]Research Department, Neuropsychopharmacology Unit, Complejo Hospitalario Universitario de Albacete, Albacete, Spain. [6]Centro Tecnológico de la Carne de Galicia, rúa Galicia n° 4, Parque Tecnológico de Galicia, San Cibrao das Viñas, 32900 Ourense, Spain. [7]Área de Tecnología de los Alimentos, Facultad de Ciencias de Ourense, Universidad de Vigo, 32004 Ourense, Spain. [8]Institute of Antler Science and Product Technology, Changchun Sci-Tech University, Corner of Pudong Road and Suzhou Bei Street, Changchun City, Jilin, P. R. China. [9]Department of Neurology, Complejo Hospitalario Universitario de Albacete, 02006 Albacete, Spain.

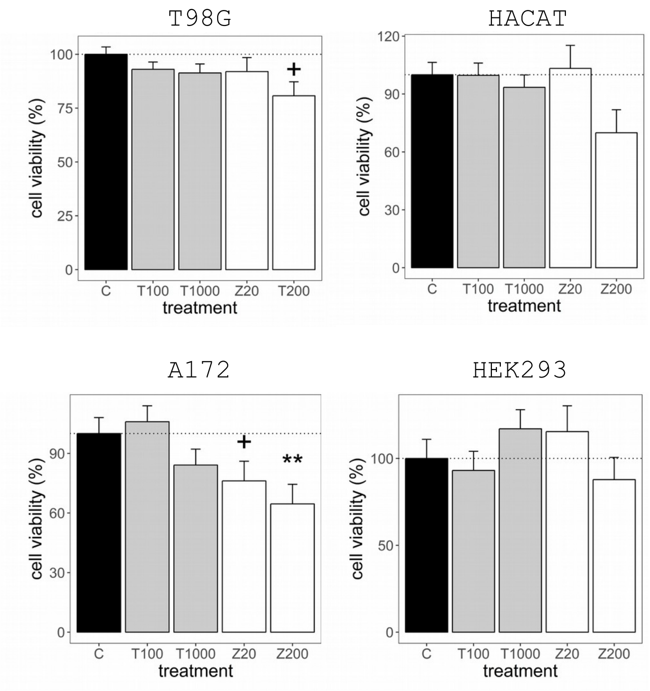

Fig S1 Cell Viability Assay**.** Cytotoxicity in T98G, HACAT, A172, and HEK293 cells after 24 h incubation with DAV extract and TMZ. Values are shown in µg/ml. T=Tip portion; Z=TMZ. Linear mixed models to assess the effects of treatments were used as described in Fig 2. Data are means ± SEM (n=2).

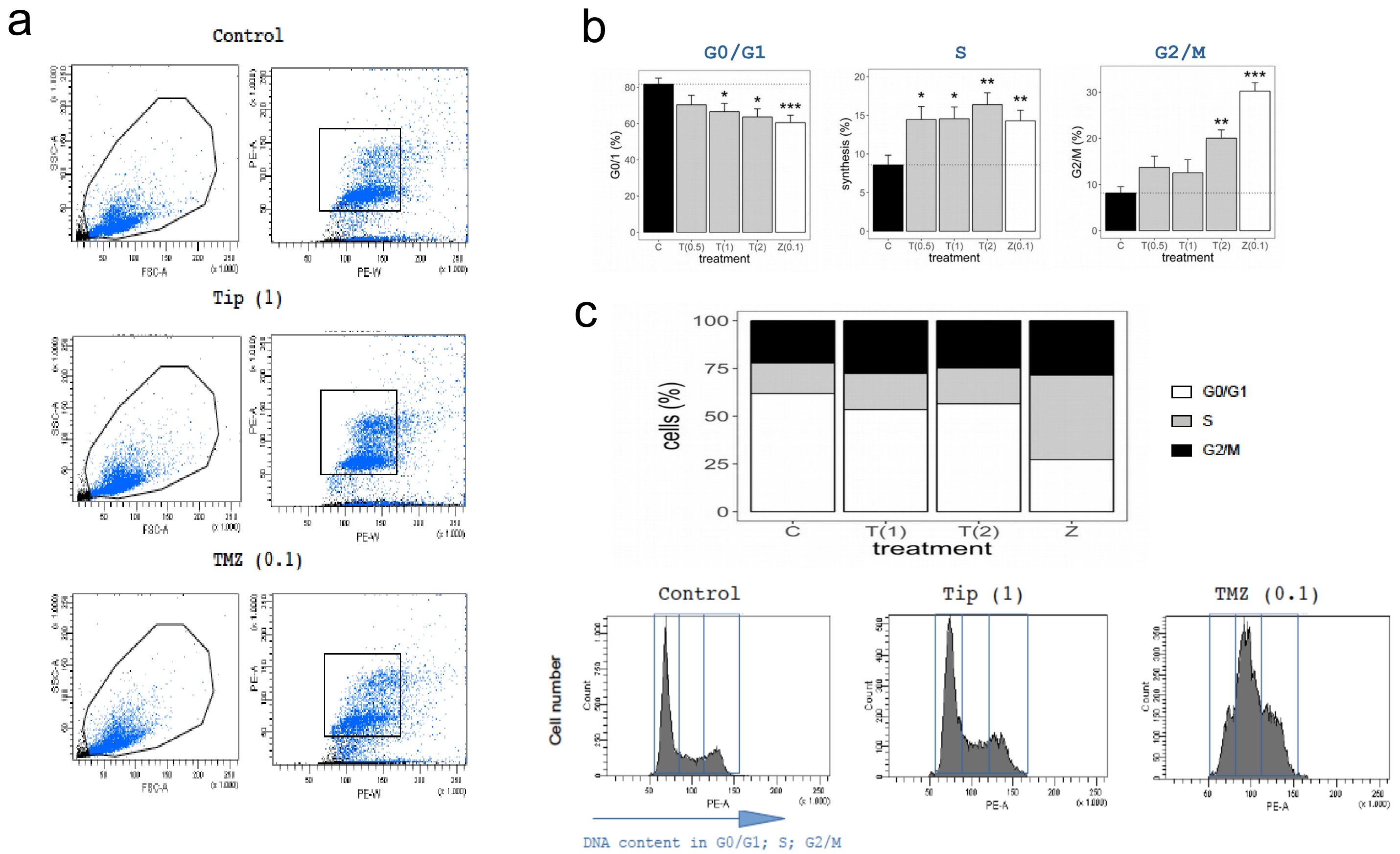

Fig. S2 Cell Cycle Analysis. **a** Flow Cytometry analysis of T98G cells. Gate SSC-A *vs* FSC-A identifies cells (left) and gate PE-A vs PE-W discriminates doublets 2xW (right). **b** T98G cell percentages for each cell cycle phase. Compilation of this percentages is shown in a bar graph in figure 5. **c** Bar graphs illustrate results of HEK293 cell cycle analysis, indicating the percentage of cells in G0/G1, S, and G2/M cell cycle phases after treatment with DAV at 1 and 2 mg/ml and with TMZ at 0.1 mg/ml. HEK293 cell line were stained with propidium iodide at 24 hours following initiation of treatment. Representative histogram plots of cell cycle distribution are shown beside. Values in brackets stand for compound concentration in mg/ml. Linear mixed models to assess the effects of treatments were used as described in Fig 1. Data are means ± SEM (n=2).

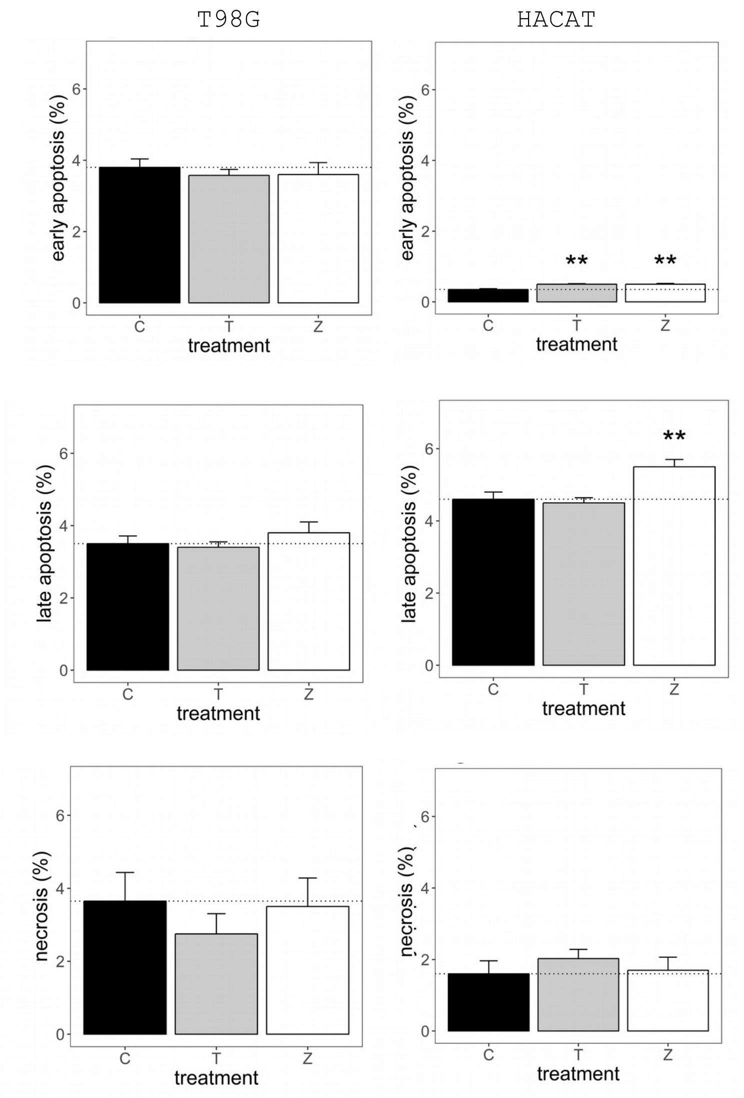

Fig. S3 Percentage of T98G and HACAT early apoptotic cells (upper), late apoptotic cells (middle), and necrotic cells (lower) following treatment with DAV and TMZ at 1 mg/ml and 0.1 mg/ml at 24h, respectively. Linear mixed models to assess the effects of treatments were used as described in Fig 1. Data are means ± SEM (n=2).

random effects					
groups (n = 2)	sd				
assay (intercept)	0				
residual (n = 29)	21.6				
fixed effects	estimate	se	df	t-value	*p*
control (Intercept)	100	9.66	22	10.35	<0.0001
Mid1000	-15.5	13.08	22	-1.188	0.247
Tip1000	-46.1	15.78	22	-2.923	0.008
Mid100	4.7	14.49	22	0.324	0.749
Tip100	-2.6	14.49	22	-0.179	0.859
Mid500	-5.1	15.78	22	-0.321	0.751
Tip500	-24.5	14.49	22	-1.688	0.105
$R^2_{GLMM(m)}$	0.333				
$R^2_{GLMM(c)}$	0.333				

Table S1. Model Fig1. Estimates of the linear mixed model on cell viability assays (percentage of viability) of the cytotoxicity of DAV extracts (tip and middle) at concentrations of 100, 500, 1000 µg/ml on T98G cell line. $R^2_{GLMM(m)}$ marginal explained variance. $R^2_{GLMM(c)}$ conditional explained variance. Contrasts of control against treatment levels are shown in Figure Fig 1a.

random effects					
groups (n = 7)	sd				
sampling points	24334				
egf	38743				
tip	14141				
residual (n = 308)	6872				
fixed effects	estimate	se	df	t-value	p
control (Intercept)	136384.9	13569.48	5	10.05	0.0001
time	-6329.2	1060.36	286.9	-5.97	<0.0001
egf	-5131.8	22941.36	5.6	-0.22	0.831
tip	-5964.7	13369.86	34	-0.45	0.658
0-6h	25819.6	9013.67	286.9	2.86	0.004
time × egf	2111.3	1499.58	286.9	1.41	0.16
time × tip	582.3	1364.37	286.9	0.43	0.67
time × 0-6h	-4602.6	1176.37	286.9	-3.91	0.0001
egf × 0-6h	16300.9	12747.26	286.9	1.28	0.202
tip × 0-6h	-21734.6	11567.75	286.9	-1.88	0.061
time × egf × 0-6h	-3067	1663.63	286.9	-1.84	0.066
time × tip × 0-6h	4244.1	1507.63	286.9	2.82	0.005
$R^2_{GLMM(m)}$	0.491				
$R^2_{GLMM(c)}$	0.962				

Table S2. Model Fig 4. Estimates of the linear mixed model on the rate of scratch healing ($\mu m^2 h^{-1}$) of cells T98 under different treatments. control; EGF at 10^{-5} mg/ml ; tip: DAV extract at 1mg/mL. Rates of the scratch assay were assessed within two phases, between 0 and 6 hours (0-6h) and between 6 and 10 h, because there was evidence that there was a change of rate at time = 6 h. $R^2_{GLMM(m)}$ marginal explained variance. The levels of reference are: time series 6 – 10 h, and treatment = control. $R^2_{GLMM(c)}$ conditional explained variance. The regression lines of this model are shown in Fig. 4.

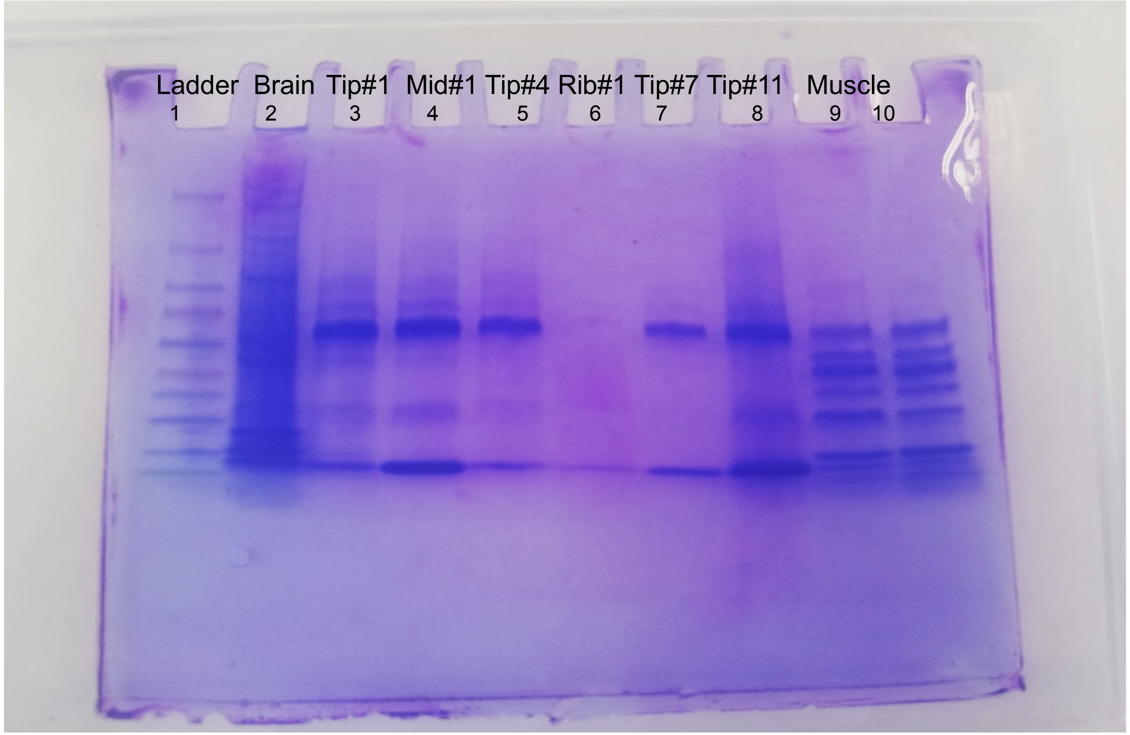

Uncropped gel, Fig 1. **1**. Ladder. **2**. Total protein from a mouse brain used as a control. **3**. DAV extract from the tip portion of deer#1. **4.** DAV extract from the middle portion of deer#1. **5**. DAV extract from the tip portion of deer#4. **6**. Rib extract from deer#1. **7**. DAV extract from the tip portion of deer#7 **8.** DAV extract from the tip portion of deer#11. **9, 10**. Deer muscle

Legends for the supplementary videos

Supp. V1. Video with HACAT cells in a scratch assay without treatment (control). Images were captured every 30 min for 15 h.

Supp. V2. Video with HACAT cells in a scratch assay treated with 1 mL EGF at 10 ng/mL. Images were captured every 30 min for 15 h.

Supp. V3. Video with HACAT cells in a scratch assay treated with 1 mL DAV extract at 1 mg/mL. Images were captured every 30 min for 15 h.

Supp. V4. Video withT98G cells in a scratch assay without treatment (control). Images were captured every 30 min for 15 h.

Supp. V5. Video with T98G cells in a scratch assay treated with 1 mL EGF at 10 ng/mL. Images were captured every 30 min for 15 h.

Supp. V6. Video with T98G cells in a scratch assay treated with 1 mL DAV extract at 1 mg/mL. Images were captured every 30 min for 15 h.
